# Epigenetic therapies by targeting aberrant histone methylome in AML: molecular mechanisms, current preclinical and clinical development

**DOI:** 10.1038/onc.2016.315

**Published:** 2016-09-05

**Authors:** C T Tsai, C W E So

**Affiliations:** 1 Leukaemia and Stem Cell Biology Group, Division of Cancer Studies, Department of Haematological Medicine, King's College London, Denmark Hill Campus, Rayne Institute, London, UK

## Abstract

While the current epigenetic drug development is still largely restricted to target DNA methylome, emerging evidence indicates that histone methylome is indeed another major epigenetic determinant for gene expression and frequently deregulated in acute myeloid leukaemia (AML). The recent advances in dissecting the molecular regulation and targeting histone methylome in AML together with the success in developing lead compounds specific to key histone methylation-modifying enzymes have revealed new opportunities for effective leukaemia treatment. In this article, we will review the emerging functions of histone methyltransferases and histone demethylases in AML, especially MLL-rearranged leukaemia. We will also examine recent preclinical and clinical studies that show significant promises of targeting these histone methylation-modifying enzymes for AML treatment.

## Introduction

Although intensive chemotherapy combined with transplantation of haematopoietic stem cells have considerably improved the outcomes in certain subgroups of younger leukaemia patients, acute myeloid leukaemia (AML) as the most common type of acute leukaemia in adults remains highly fatal and around 80% of patients aged over 60 succumb to the disease or the highly toxic treatment regimens.^1, 2^ AML is a heterogeneous group of diseases that can be further classified into different subtypes according to their distinctive genetic mutations with variable prognostic significances. In spite of the large arrays of mutations reported in AML, most of them specifically affect transcription factors or key components of epigenetic machinery. Importantly, chimeric fusions that are believed to be the initiating events in translocation leukaemia almost always involve transcription/epigenetic factors.^3^ Among them is the mixed lineage leukaemia gene (*MLL*) that associates with a very poor prognosis and treatment resistant.^4^ Similar mutational profiles affecting transcriptional and epigenetic machinery have also been reported in normal karyotype AML, where DNA methyltransferase 3A and NPM are found to be the early events and persist during relapses,^5, 6, 7^ consistently indicating the importance of transcriptional deregulation in AML pathogenesis. In spite of our advance in understanding the genetics of AML, very little has been translated into the clinics and we are still using the same highly toxic and rather ineffective chemotherapies developed over a half-century ago. Therefore, there is an urgent need to identify novel venues for more potent and effective drug development to tackle this formidable disease. While development of small-molecule inhibitors to transcription factors remains technically challenging, the recent discoveries of critical function of epigenetic modifying enzymes with structurally rigid motifs and/or catalytic domains in AML pathogenesis have fuelled the enthusiasm to target these intractable oncogenic events. In this review, we will focus on some of the latest preclinical and clinical development of epigenetic therapy in AML, in particular, those involve *MLL* gene rearrangements.

## Epigenetic therapies targeting DNA methylation and histone acetylation in AML

The term epigenetics refers to alternations of gene expression that are inheritable after cell division without any changes in DNA sequence.^8^ In addition to DNA methylation, an increasing number of epigenetic modifications on histones, including acetylation, methylation and ubiquitination, have been identified and are frequently deregulated in AML,^9, 10^ resulting in repression of tumour suppressor genes and/or activation of oncogenic pathways.^11^ Aberrant DNA methylation and histone acetylation are two most ancient and better characterized epigenetic changes. DNA methylation, leading to gene silencing, is prevalent in cancers including leukaemia, and has been the target for cancer therapy since the FDA approval of DNA methyltransferase inhibitors (DNMTi), azacytidine and decitabine for the treatment of myelodysplastic syndrome and certain AML.^12^ Although AML patients aged over 65 years who treated with DNMTi did not show significantly longer overall survival (OS) as compared with conventional care regimen, azacytidine and decitabine displayed safety and better clinical efficacy in patients with unfavourable cytogenetics or myelodysplasia-related changes, indicating that they may be preferable therapies for these ‘difficult-to-treat' AML population.^13, 14^ In addition to DNMTi, a number of pan-histone deacetylase inhibitors inducing chromatin remodelling and re-expression of tumour suppressor genes are also designed and utilized in AML treatment.^15^ While single-agent therapy was reported only having modest clinical activity, combination of histone deacetylase inhibitors with DNMTi (decitabine, complete remission: 31%) or with Ara-c (cytarabine, complete remission: 78%, OS: 82 weeks) in clinical trials appeared to be synergistic and profoundly improved responses.^16, 17^ Although these early endeavours on heterogeneous myeloid malignancies have demonstrated the safety and potential therapeutic values of targeting epigenetic machinery in clinical settings, it also urges the need of better understanding of the epigenetic regulation and exploring novel critical targets for effective AML treatment. To overcome the problems associated with genetic heterogeneity that may, in part, account for the poor efficacy of DNMTi or histone deacetylase inhibitors in the clinics, recent studies focusing on systematic analyses of leukaemia carrying chimeric transcription factors or specific mutations affecting histone methylation-modifying enzymes provide important insights and novel tractable targets for epigenetic therapies in AML.

## The role of histone methyltransferases in AML

Depending on the position and nature of the methylated residues, histone methylation can have positive as well as negative impacts on gene expression.^18^ Histone methylation features epigenetic modification in which lysine and arginine residues can be mono-(me1), di-(me2) or even tri-(me3) methylated (for lysine only). In general, methylation of histone 3 lysine 4 (H3K4), lysine 36 (H3K36), lysine 79 (H3K79), as well as asymmetric dimethylation of histone 4 arginine 3 (H4R3) activates gene expression; whereas methylation on other sites like histone 3 lysine 9 (H3K9), lysine 27 (H3K27), histone 4 lysine 20 (H4K20) and symmetric dimethylation of H4R3 associates with transcription repression.^18, 19^ H3K4me3 and H3K27me3 that define bivalent marks are predominately mediated by two master epigenetic regulators, trithorax group proteins with HRX/MLL as the founding member and polycomb group proteins with EZH1/2 as the catalytic subunits of polycomb repressor complex 2 (PRC2) in mammalian cells.^20^ Intriguing, the key components of both trithorax group and polycomb group complexes are frequently mutated in AML.

Investigating the association of chromosome 7q abnormalities in myeloid malignancy has revealed an important role of EZH2 in leukaemogenesis. EZH2 regulates expression of numerous genes critical for stem cell renewal by mediating a H3K27 methylation.^21^ EZH2 mutations were found in 9 of 12 patients with chromosome 7q acquired uniparental disomy, and the majority of EZH2 mutations resulted in loss of its H3K27 methyltransferase activity,^22^ which is in contrast with its gain of function mutation in B-cell lymphoma.^23^ Deletion of EZH2 was able to induce a myelodysplastic syndrome-like disease in a mouse model, suggesting the tumour suppressor function of EZH2 in certain myeloid malignancies.^24, 25, 26^ On the other hand, loss-of-function mutations of ASXL1, another polycomb group protein, are usually associated with unfavourable OS and poor complete remission rate in AML.^27^ Although its molecular function in leukaemic transformation is still unclear, depletion of ASXL1 showed loss of PRC2-mediated H3K27 trimethylation and led to upregulation of *HOXA* genes including *HOXA5* and *HOXA9*. On the contrary, overexpression of ASXL1 resulted in a global increase of H3K27 me2/3 and suppression of *HOXA* genes and cell growth. ASXL1 can interact with EZH2 in human leukaemic cells, and loss of ASXL1 resulted in displacement of PRC2 from *HOXA* loci.^28^ ASXL1 may also collaborate with BAP1, loss of which led to a myelodysplastic syndrome-like syndrome in a mouse model, to deubiquitinate H2AK119 at polycomb group targets.^29, 30^ Haematopoietic-specific knockout of ASXL1 profoundly impaired cell differentiation and induced myeloid dysplasia and erythroid dysplasia in knockout mice. Furthermore, transplantation of ASXL1-null LSK cells or bone marrow cells into recipient mice strikingly caused lethal myelodysplastic disorder.^31^ In addition to ASXL1, JARID2 has also been identified as an essential cofactor in promoting PRC2 recruitment to downstream targets. An acquisition of JARID2 mutation showed a positive correlation with disease progress from myelodysplastic syndrome to AML.^32^ Together, these studies reveal the critical role of EZH2 and PRC2 in malignant haematopoiesis.

**Figure 1:**
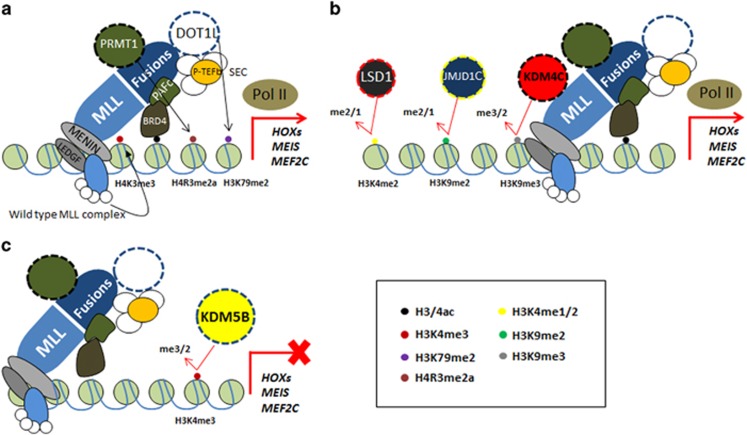
Roles of HMTs and KDMs in MLL-driven transcription. (**a**) MLL fusion complexes are assembled by recruiting a body of important components (super elongation complex, polymerase-associated factor complex, MENIN and LEDGF) to target and facilitate expression of crucial leukaemogenic genes, such as *HOXs*, *MEIS* and *MEF2C*, where HMTs (DOT1L, PRMT1 and MLL) are involved to add active methyl marks (H3K79me2/3, H4R3me2a and H3K4me3), respectively. BRD4, a histone mark reader, is essential for the recruitment of MLL fusions. (**b**) In addition to enrichment of active marks, KDMs (e.g., KDM4C, JMJD1C) on the other hand remove repressive marks (H3K9me3) to underpin the active status. Although LSD1 has been suggested to remove H3K4me1/2 marks in MLL leukaemia, its relevance to leukaemia suppression is still largely unknown. (**c**) KDM5B negatively regulates MLL target genes through demethylation of H3K4me3 active mark. Black arrows indicate methylation, whereas bent red arrows represent demethylation.

*MLL* as a master transcriptional and epigenetic regulator containing a number of functional domains including AT hook and CXXC motifs at the N-terminal and the C-terminal SET domain, which mediates specific H3K4 methylation, is predisposed to abnormal gene rearrangements resulting in a highly aggressive form of leukaemia.^33^ As a result of chromosomal translocations, chimeric MLL fusions resulting from replacement of C-terminal region of MLL including the SET domain by various fusion partners such as AF4/6/9/10, ELL and ENL can form macromolecular complexes through recruitment of a cohort of cofactors including super elongation complex (for example, positive transcription elongation factor b, MLL fusion partners such as AF4 family and AF9/ENL family), polymerase-associated factor complex, BRD3/4, MENIN and key histone methyltransferases (HMTs) (for example, DOT1L and protein arginine methyltransferases (PRMT1)) to activate gene expression programmes crucial for the transformation^18^ (Figure 1a). Identification of key aberrantly recruited HMTs by MLL fusions provide the first hint of their involvement in human cancer.^34^

DOTlL is the only lysine methyltransferase (KMT) known to be responsible for H3K79 methylation in humans. Aberrant recruitment of DOT1L specifically associates with an abnormally high level of H3K79me2 on promoters and gene bodies of MLL targets in MLL-rearranged leukaemia. The remarkable correlation of H3K79me2 and MLL targets has been referred to as a special epigenetic lesion in MLL leukaemia, implying the essentiality of H3K79 methylation for MLL-driven transcription.^35^ Inactivation of DOT1L profoundly suppressed the expression of MLL translocation-associated genes (for example, *HOXA*s and *MEIS1*) and leukaemia development^36, 37, 38, 39, 40^ (Figure 1a). Direct fusion of DOT1L to MLL was sufficient to activate transcription of *HOXAs*.^39^ Loss of DOT1L resulted in reduction of cell growth, increased differentiation and apoptosis of MLL-AF9 leukaemic cells, indicating its potential as a target for AML therapy.^38^ On the other hand, PRMT1 is the founding member of PRMTs that mediates arginine methylation on both histone (H4R3me2a) and non-histone substrates (for example, transcription factors and splicing factors). Identification of its essential function in MLL leukaemia had also provided the first evidence of PRMT involvement in human cancer.^41^ PRMT1 recruitment is required for a subset of MLL (MLL-EEN and MLL-GAS7) and non-MLL (MOZ-TIF2 and AML1-ETO) leukaemia.^41, 42, 43^ Its inhibition resulted in specific transcriptional and leukaemic suppression in MLL-rearranged and MOZ-TIF2 leukaemia.^42^ Silence of PRMT1with an short hairpin RNA (shRNA) approach attenuated the level of H4R3me2a and gene expression of HOXA9 and MEIS1, thus leading suppression of leukaemogenesis of MOZ-TIF2 and MLL-GAS7. More recently, a functional link between PRC2 and MLL leukaemia had also been proposed. EZH2 and EED, two core components of PRC2, had been shown to be required for MLL leukaemia, although the underlying mechanisms remain largely unknown.^44, 45^ While these studies highlight the importance of HMTs in MLL leukaemia, emerging evidence also reveals an equally important role of histone demethylases (HDMs) that counteract the functions of HTMs in modulating the epigenetic regulation of gene expression in both normal and cancer settings.

## The role of HDMs in AML

Protein methyltransferases (including KMTs and PRMTs indicated in the previous section) mediate methylation on specific amino acid residues, which can, however, be erased by HDMs mostly lysine demethylases (KDMs). Based on their catalytic mechanisms, KDMs can be divided into two major subgroups. The first family including KDM1A and KDM1B is also known as lysine-specific demethylase (LSD), consisting of FAD-dependent amine oxidase, which can only remove mono- and di-methyl marks.^46^ On the contrary, the second KDM family contains JmjC domain (JMJD), which relies on α-ketoglutarate, Fe(II) and oxygen as cofactors to mediate demethylation of mono-, di- and even tri-methyl-lysine residues.^47^ JMJD demethylases consist of more diverse family members and can be further divided into seven subfamilies from KDM2 to KDM8 according to their other structurally conserved domains like PHD and Tudor domains, which may also bear crucial functions in recognising/reading the histone marks.^19^

KDMs can be found differentially expressed in various cancers including leukaemia, and cooperate with transcription factors to activate or repress gene expression. LSD1 is overexpressed in MLL leukaemia and seems to have a crucial role in sustaining the oncogenic transcriptional programmes mediated by MLL fusions via an unknown mechanism (Figure 1b). LSD1 suppression by an shRNA approach led to a reduction of mouse MLL leukaemic stem cells (LSCs).^48^ Although this study suggests a requirement of H3K4 demethylase for MLL leukaemia, a recent report revealed an opposite role of H3K4 demethylase KDM5B that negatively regulated MLL LSC (Figure 1c).^49^ In this study, H3K4me2/3 but not H3K79me2 were critical for MLL LSC, and H3K4 methylation levels reduced during differentiation. Suppression of KDM5B significantly promoted disease progression, whereas its overexpression inhibited MLL leukaemia. While the reasons underlying the different results need further investigations, LSD1 on the other hand underpins retinoic acid receptor (RARa)-driven repression of myeloid differentiation-associated genes in AML through decreasing the level of H3K4me2.^50^ These results may suggest a more generic role of H3K4 methylation in AML pathogenesis, which may not be specific to MLL leukaemia. Other members of KDMs including KDM2B and JMJD1C also implicate in AML pathogenesis. H3K36me2 demethylase KDM2B that silences p15 expression was sufficient to transform haematopoietic progenitors *in vitro*, and its depletion significantly impaired HOXA9/MEIS1-driven leukaemogenesis and self-renewal of LSCs.^51^ H3K9 demethylase JMJD1C was identified as a crucial factor for the maintenance of AML expressing MLL-AF9 in an shRNA functional screen (Figure 1b). Depletion of JMJD1C inhibited cell growth and leukaemogenesis of MLL-AF9 cells by triggering apoptosis.^52^ JMJD1C had also been recently implicated in AML1-ETO-^53^ and HOXA9-mediated leukaemias.^54^ JMJD1C was identified as a coactivator in AETFC, a complex formed by AML1-ETO, where JMJD1C maintained low level of H3K9me2, hence enhancing gene expression of AML1-ETO targets. Knockdown of JMJD1C compromised the ability of AML1-ETO to inhibit cell differentiation and impaired colony formation.^53^ JMJD1C also interacted with HOXA9 to modulate the downstream genes critical for self-renewal of LSCs. Loss of JMJD1C profoundly affected leukaemic transformation driven by HOXA9, indicating yet another KDM family member with a more generic function in AML pathogenesis.^54^

## Crosstalk between HMTs and HDMs in AML

**Figure 2:**
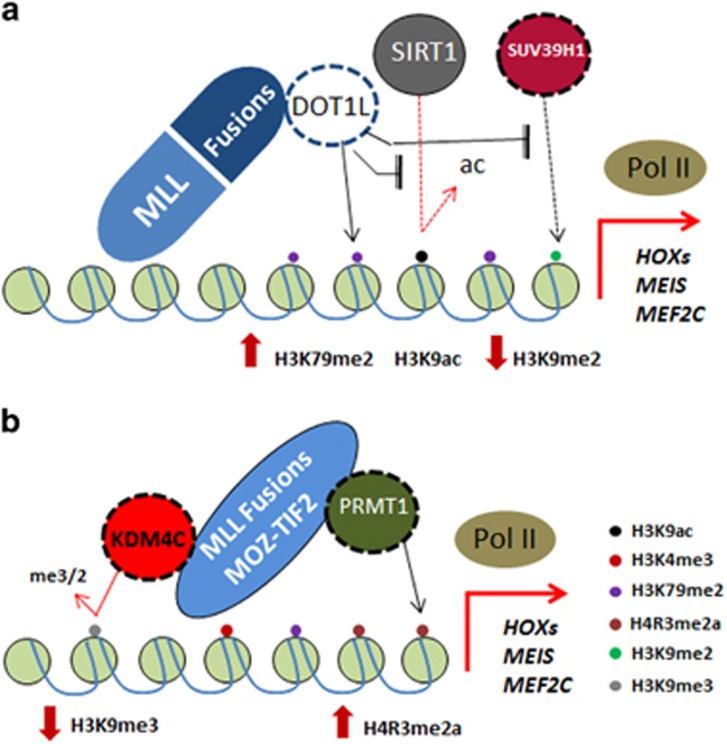
Crosstalk between HMTs and HDMs in MLL-driven transcription. (**a**) When DOT1L is recruited by MLL fusions, it confers H3K79me2 active mark, which may further expel SUV39H1 and SIRT1, hence leading to a reduction in H3K9me2 repressive mark but an increase in H3K9ac activation mark. (**b**) After binding to MLL fusions and MOZ-TIF2, PRMT1 and KDM4C cooperate to maintain the activation of MLL-driven transcription by conferring a high level of H4R3me2a, but a low level of H3K9me3 repressive mark.

Although the above reports have directly implicated individual HMT and HDM in AML pathogenesis, their mode of actions and underlying mechanisms remain largely unknown. Recent studies exploring the functional crosstalk between HMTs and HDMs have shed lights into the intricate molecular regulation of aberrant histone methylome in AML. It has been demonstrated that the chromatin localisation of SIRT1, a H3K9 deacetylase, and SUV39H1, a H3K9 methyltransferase, may be disrupted by DOT1L (Figure 2a). After inhibition of DOT1L, SIRT1 and SUV39H1 bound to MLL targets such as *HOXA7* and *MEIS1* and exerted their function to establish a heterochromatin-like state, in which the level of H3K9me2 but not H3K79me2 was kept considerably high. Deletion of SIRT1 or SUV39H1 significantly desensitised MLL-AF9 leukaemic cells to DOT1L inhibition, whereas pharmacological activation of SIRT1 by SRT1720 strikingly improved the *in vivo* efficacy of EPZ4777, a DOT1L inhibitor, demonstrating a critical function of this crosstalk in regulating DOT1L inhibitor sensitivity.^55^ To search for novel epigenetic regulators that cooperate with PRMT1 in AML pathogenesis, KDM4C was identified to specifically interact with MLL fusions and MOZ-TIF2 to remove H3K9me3 repressive mark.^42^ Together with PRMT1, KDM4C co-regulated the epigenetic programmes for transcriptional deregulation and cellular transformation by increasing the H4R3me2a active mark but attenuating H3K9me3 repressive mark on MLL downstream targets such as *HOXA9* (Figure 2b). Similar to PRMT1, shRNA-mediated suppression of KDM4C resulted in repression of MLL downstream gene expression programmes, attenuation of leukaemogenesis and a significant improvement of OS in mouse and humanized models, revealing the requirement of the presence of both epigenetic modifying enzymes for the oncogenic functions of MLL fusions. As KDM4C binds to MLL N-terminus region, KDM4C is also required for leukaemia induced by other MLL fusions independent on PRMT1, suggesting a much broader function of KDM4C in maintaining aberrant epigenetic networks in MLL leukaemia. Interestingly, a recent study suggested a potential redundant function among KDM4 family members in AML using a tamoxifen inducible knockout approach.^56^ Although characterisation of the actual genotype on Kdm4c knockout leukaemic cells was not performed, genetic escape from *in vivo* deletion of Kdm4 family seems to be a common theme in all the resultant leukaemia with genotyping results, which was in line with the requirement of a very high dose of tamoxifen to achieve even *in vitro* deletion. Nevertheless, these studies consistently indicate critical functions of KDM4 family in acute leukaemogenesis.

## The role of KDM in treatment response

**Figure 3:**
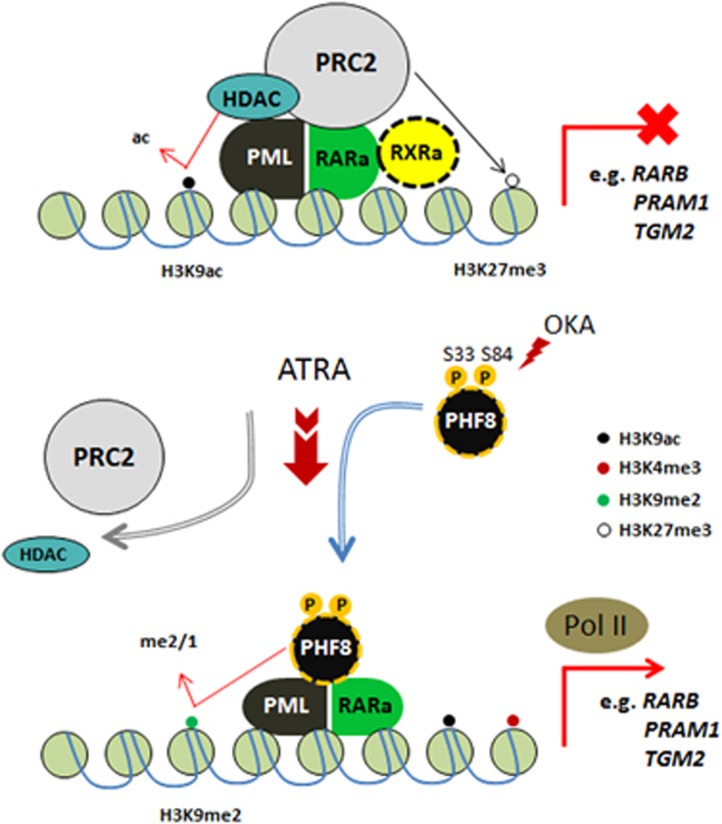
Roles of KDMs in ATRA therapeutic response. PML–RARa forms a repressor complex with RXRa, HDACs and PRCs to inhibit the expression of myeloid differentiation-associated genes. In non-APL, LSD1 is also recruited to further remove H3K4me2/1, contributing to a more stable repressive status (not shown in figure). ATRA treatment results in a conformational change of PML–RARa, leading to dissociation of HDACs and PRCs, and recruitment of phosphorylated PHF8 to confer transcriptional activation. Activation of PHF8 by okadaic acid (OKA) may sensitise refractory APL cells to ATRA treatment.

In addition to disease progression, KDM has also been implicated in governing treatment response in acute promyelocytic leukaemia (APL) driven by RARa fusions. APL is the only AML subgroup with a well-established targeted therapy where all trans retinoic acid (ATRA) can induce transcriptional de-repression and leukaemic differentiation. In spite of success in identifying repressor complexes associated with RARa fusions, the identity of the activator being recruited by the fusions upon ATRA treatment had remained elusive. To search for such a regulator, PHF8 (KDM7B), a H3K9 demethylase, was found to specifically interact with RARa fusion proteins to remove H3K9me2 repressive mark upon ATRA treatment^57^ (Figure 3). ATRA treatment results in a conformation change of RARa fusions, leading to dissociation of corepressors such as histone deacetylase and PRC2. PHF8 acts as a critical sensor for ATRA treatment response, which is dependent on both the enzymatic activity and the phosphorylation status of two critical serine residues of PHF8 that partly determine its chromatin localisation. Genetic or pharmacological activation of PHF8 sensitized ATRA refractory cells to the treatment, whereas its suppression conferred resistance to APL cells. These findings for the first time directly implicate the activity of KDM in governing AML treatment responses, and reveal a novel therapeutic venue to overcome treatment resistant.^58^ In addition to PHF8, LSD1 is also involved in the repressive machinery of RAR fusions (Figure 3). Inhibition of LSD1 could increase the level of H3K4me2 on the promoters of myeloid differentiation-associated genes and triggered ATRA therapeutic response in non-APL AML. Although ATRA exhibited little effects in non-APL AML, combination of ATRA and the LSD1 inhibitor TCP remarkably initiated cell differentiation of non-APL AML and reduced colony formation and engraftment of AML.^50^

## Histone methylome as an emerging therapeutic target

Given the critical functions of histone methylome in AML, the first HMT inhibitor targeting DOT1L, EPZ4777^59^ and its second-generation derivative, EPZ5676^60^ have been developed and tested for suppressing MLL leukaemia. Both compounds showed selective inhibitory effects on H3K79 methylation and cells bearing MLL fusions (Table 1). Continuous infusion of DOT1L inhibitors significantly prolonged the OS in murine models with MLL leukaemia,^59, 60^ leading to the first clinical trial of HMT inhibitors in AML. However, the undesirable pharmacokinetic characteristics of the DOT1L inhibitors may limit their clinical development,^60^ and can be partly responsible for the rather modest clinical responses in their early trial results. On the other hand, a PRMT1 inhibitor, AMI-408 could also significantly extend disease latency and OS in mouse models carrying MLL-GAS7 fusion or MOZ-TIF2.^42^ Similarly to DOT1L inhibitors, the efficacy of PRMT1 inhibitors in leukaemia suppression was far inferior to those by genetic or shRNA approaches, indicating the need to improve the pharmacokinetics of these early phase inhibitors. Studies also reported prolonged OS and reduced tumour burden in MLL-AF9 leukaemia model by targeting of a H3K27 methyltransferase EZH2. The EZH2 inhibitor DZNep triggered apoptosis of AML cells through reactivating TXNIP, leading to the accumulation of reactive oxygen species.^61^ Depletion of EZH2 or pharmacological inhibition of EZH1/2 by a small-molecule UNC1999 upregulated PRC2 target genes such as *p16* and *p19* in MLL leukaemic cells.^62, 63^ There are also highly effective EZH2 inhibitors such as GSK126 and EPZ5687 for diffuse large B-cell lymphoma.^64, 65^ GSK126 and stapled hydrocarbon peptide targeting EZH/EED interaction have been tested in parallel and shown strong suppression of *in vitro* MLL leukaemia cell growth, although their *in vivo* efficacy has yet to be demonstrated.^66^

Similarly, while very limited *in vivo* data has been shown, a monoamine oxidase inhibitor, tranylcypromine (TCP) alone or in combination with ATRA has been used to suppress LSD1 activity in MLL^48^ or non-MLL leukaemia *in vitro*^50^ (Table 1), respectively. A TCP derivative, GSK2879552 has entered phase I clinical trials for the treatment of relapsed AML (ClinicalTrials.gov identifier: NCT02177812), however TCP exhibited severe toxicity at efficacious doses in preclinical models, so it is possible that the TCP may result in broad toxicity, especially in central nervous system.^67^ Recently, a non-monoamine oxidase inhibitor SP2509 with similar efficacy but lower general toxicity as compared with TCP was developed (Table 1). SP2509 blocked the interaction between LSD1 and the co-repressor CoREST, thus leading to a permissive increase in H3K4me3 on target genes such as *p21*, *p27* and *CCAAT*/enhancer-binding protein. SP2509 was able to effectively suppress colony formation, induced cell differentiation and triggered apoptosis of AML cells with mutant NPM1 but not MLL fusions.^68^ However, while inhibition of LSD1 showed a significant efficacy in a mouse xenograft model, it could only consistently translate into extended OS when it was a combination with PS, a pan-histone deacetylase inhibitor.^68^ It is noted that all the above epigenetic targets, in particular, DOT1L and LSD1 are absolutely essential for normal development and haematopoietic stem cells, which may limit the application of effective dose in patients and, therefore, a combination approach with lower dose may be beneficial. On the other hand, KDM4C is largely dispensable for normal development and its complete deletion does not lead to any significant phenotypes in the mouse model. Consistently, an early phase KDM4C inhibitor, SD70 displayed an excellent therapeutic effect on AML expressing MOZ-TIF2 and MLL fusions^42^ (Table 1). Pharmacological inhibition of KDM4 effectively attenuated leukaemogenesis *in vivo* and extended OS in both mouse and humanized models with primary human MLL leukaemia. Remarkably, SD70 is quite well tolerated and has limited toxicity in these preclinical models, highlighting its therapeutic potentials for AML treatment.

## Prospective

Transcriptional deregulation plays the key role in acute leukaemogenesis and, probably, treatment responses. The emerging functions of various epigenetic modifying enzymes of histone methylome in AML pathogenesis have provided unique opportunities to target this group of dismal disease, in which its treatment regime has not really changed for decades. In addition to the histone mark writers and erasers, it is also possible to target readers that are essential to recognize these specific histone marks for aberrant gene expression and transformation. It has been proposed that WDR5, one of the main components of MLL complexes essential for MLL histone methyltransferase activity, recognises H3K4me and presents the K4 side chain for further methylation by MLL.^49, 69, 70^ Blocking MLL1–WDR5 interaction by a small-molecule inhibitor MM-401 specifically reduced levels of H3K4me at *HOXA* loci, induced myeloid differentiation and triggered apoptosis of mouse MLL-AF9 leukaemic cells.^71^ In addition to WRD5 family, significant progress has been made to target bromodomain that recognizes acetyl-lysine marks. One of the best examples is the potential targeting of BRD family in MLL leukaemia. Genetic or pharmacological inhibition of BRD3/4 by I-BET151 (GSK1210151A) or JQ1 led to the suppression of BCL-2, Myc and CDK6 in leukaemic cells, and displayed outstanding efficacy against mouse and human leukaemia cells driven by MLL fusions *in vitro* and *in vivo* (Table 1).^72, 73^ Although rapid development of drug resistant in preclinical models, in part, due to activation of canonical Wnt/b-catenin signalling may pose a threat for effective treatments by BRD inhibitors,^74, 75^ these studies provide an important proof-of-principle data showing the feasibility of targeting protein–protein interaction involved in epigenetic regulation for leukaemia treatment. Similar principles will likely be applicable to other readers involved in histone methylome such as chromodomain and PHD domain. Future studies in dissecting the molecular regulation of critical histone methylome writers, readers and erasers will open up a promising venue for the development of next-generation effective AML treatments.

## Figures and Tables

**Table 1:** Emerging epigenetic drugs for AML treatment

*Compound*	*Target*	*Mechanism*	*Development*
DOT1L inhibitor	H3K79 methyltransferase DOT1L	Depletion of H3K79me2, leading to repression of MLL fusion targets	Preclinical EPZ4777^59^ Phase I clinical trial EPZ5676 (NCT01684150, MLL leukaemia)
PRMT1 inhibitor	H4R3 methyltransferase PRMT1	Depletion of H4R3me2a, leading to repression of MLL fusion targets	Preclinical AMI-408^42^
EZH2 inhibitor	H3K27 methyltransferase EZH2	Suppression of H3K27me3, leading to de-repression of polycomb targets (for example, CDKN2A, TXNIP)	Preclinical DZNep^61^ UNC1999^63^
MOA inhibitor TCP, TCP derivatives	H3K4 demethylase KDM1A (LSD1)	Unknown mechanism in AML enhances H3K4me1/2?	Clinical trials GSK2879552 (NCT02177812, refractory AML) ORY-1001 (EudraCT 2013-002447-29, refractory acute leukaemia)
Non-MOA inhibitor	H3K4 demethylase KDM1A (LSD1)	Blocking the interaction of LSD1 and CoREST	Preclinical SP2509^68^
KDM4C inhibitor	H3K9 demethylase KDM4C (JMJD2C	Increase in H3K9me3, leading to repression of MLL fusion or MOZ-TIF2 targets	Preclinical SD70^42^
BET inhibitor	Bromodomain-containing proteins, BRD family	Displacement of BRD family from chromatin	Phase I clinical trials OTX015 (NCT01713582, acute leukaemia and various haematological malignancies) CPI-0610 (NCT02158858, acute leukaemia and MDS) GSK525762 (NCT01943851, relapsed haematological malignancies)

Abbreviations: AML, acute myeloid leukaemia; LSD1, lysine-specific demethylase; MDS, myelodysplastic syndrome; MLL, mixed lineage leukaemia; PRMT1, protein arginine methyltransferases.

## References

[bib1] Burnett A, Wetzler M, Lowenberg B. Therapeutic advances in acute myeloid leukemia. J Clin Oncol 2011; 29: 487–494. 21220605 10.1200/JCO.2010.30.1820

[bib2] Ferrara F, Schiffer CA. Acute myeloid leukaemia in adults. Lancet 2013; 381: 484–495. 23399072 10.1016/S0140-6736(12)61727-9

[bib3] Zeisig BB, Kulasekararaj AG, Mufti GJ, So CW. SnapShot: acute myeloid leukemia. Cancer Cell 2012; 22: 698–698.e1. 23153541 10.1016/j.ccr.2012.10.017

[bib4] Zeisig BB, So CW Cellular and molecular basis of KMT2A/MLL leukaemias: from transformation mechanisms to novel therapeutic strategies. In: Rowley JD, Le Beau MM, Rabbitts TH (eds). Chromosomal Translocations and Genome Rearrangements in Cancer. Springer: NY, USA, 2016, pp 223–250.

[bib5] Shlush LI, Zandi S, Mitchell A, Chen WC, Brandwein JM, Gupta V et al. Identification of pre-leukaemic haematopoietic stem cells in acute leukaemia. Nature 2014; 506: 328–333. 24522528 10.1038/nature13038 PMC4991939

[bib6] Kronke J, Bullinger L, Teleanu V, Tschurtz F, Gaidzik VI, Kuhn MW et al. Clonal evolution in relapsed NPM1-mutated acute myeloid leukemia. Blood 2013; 122: 100–108. 23704090 10.1182/blood-2013-01-479188

[bib7] Hou HA, Kuo YY, Liu CY, Chou WC, Lee MC, Chen CY et al. DNMT3A mutations in acute myeloid leukemia: stability during disease evolution and clinical implications. Blood 2012; 119: 559–568. 22077061 10.1182/blood-2011-07-369934

[bib8] Egger G, Liang G, Aparicio A, Jones PA. Epigenetics in human disease and prospects for epigenetic therapy. Nature 2004; 429: 457–463. 15164071 10.1038/nature02625

[bib9] Greenblatt SM, Nimer SD. Chromatin modifiers and the promise of epigenetic therapy in acute leukemia. Leukemia 2014; 28: 1396–1406. 24609046 10.1038/leu.2014.94 PMC5555411

[bib10] Wouters BJ, Delwel R. Epigenetics and approaches to targeted epigenetic therapy in acute myeloid leukemia. Blood 2016; 127: 42–52. 26660432 10.1182/blood-2015-07-604512

[bib11] Baylin SB, Jones PA. A decade of exploring the cancer epigenome - biological and translational implications. Nat Rev Cancer 2011; 11: 726–734. 21941284 10.1038/nrc3130 PMC3307543

[bib12] Navada SC, Steinmann J, Lubbert M, Silverman LR. Clinical development of demethylating agents in hematology. J Clin Invest 2014; 124: 40–46. 24382388 10.1172/JCI69739 PMC3871232

[bib13] Dombret H, Seymour JF, Butrym A, Wierzbowska A, Selleslag D, Jang JH et al. International phase 3 study of azacitidine vs conventional care regimens in older patients with newly diagnosed AML with >30% blasts. Blood 2015; 126: 291–299. 25987659 10.1182/blood-2015-01-621664 PMC4504945

[bib14] Huls G. Azacitidine in AML: a treatment option? Blood 2015; 126: 283–284. 26185114 10.1182/blood-2015-06-648071

[bib15] Quintas-Cardama A, Santos FP, Garcia-Manero G. Histone deacetylase inhibitors for the treatment of myelodysplastic syndrome and acute myeloid leukemia. Leukemia 2011; 25: 226–235. 21116282 10.1038/leu.2010.276

[bib16] Kirschbaum M, Gojo I, Goldberg SL, Bredeson C, Kujawski LA, Yang A et al. A phase 1 clinical trial of vorinostat in combination with decitabine in patients with acute myeloid leukaemia or myelodysplastic syndrome. Br J Haematol 2014; 167: 185–193. 25040094 10.1111/bjh.13016

[bib17] Garcia-Manero G, Tambaro FP, Bekele NB, Yang H, Ravandi F, Jabbour E et al. Phase II trial of vorinostat with idarubicin and cytarabine for patients with newly diagnosed acute myelogenous leukemia or myelodysplastic syndrome. J Clin Oncol 2012; 30: 2204–2210. 22585696 10.1200/JCO.2011.38.3265 PMC4879705

[bib18] Cheung N, So CW. Transcriptional and epigenetic networks in haematological malignancy. FEBS letters 2011; 585: 2100–2111. 21477587 10.1016/j.febslet.2011.03.068

[bib19] Labbe RM, Holowatyj A, Yang ZQ. Histone lysine demethylase (KDM) subfamily 4: structures, functions and therapeutic potential. Am J Transl Res 2013; 6: 1–15. 24349617 PMC3853420

[bib20] Lau PNL, So CW Polycomb and trithorax factors in transcriptional and epigenetic regulation. In: Huang S, Litt MD, C.A. B (eds). Epigenetic Gene Expression and Regulation. Elsevier: MA, USA, 2015, pp 63–69.

[bib21] Bracken AP, Helin K. Polycomb group proteins: navigators of lineage pathways led astray in cancer. Nat Rev Cancer 2009; 9: 773–784. 19851313 10.1038/nrc2736

[bib22] Ernst T, Chase AJ, Score J, Hidalgo-Curtis CE, Bryant C, Jones AV et al. Inactivating mutations of the histone methyltransferase gene EZH2 in myeloid disorders. Nat Genet 2010; 42: 722–726. 20601953 10.1038/ng.621

[bib23] Morin RD, Johnson NA, Severson TM, Mungall AJ, An J, Goya R et al. Somatic mutations altering EZH2 (Tyr641) in follicular and diffuse large B-cell lymphomas of germinal-center origin. Nat Genet 2010; 42: 181–185. 20081860 10.1038/ng.518 PMC2850970

[bib24] Muto T, Sashida G, Oshima M, Wendt GR, Mochizuki-Kashio M, Nagata Y et al. Concurrent loss of Ezh2 and Tet2 cooperates in the pathogenesis of myelodysplastic disorders. J Exp Med 2013; 210: 2627–2639. 24218139 10.1084/jem.20131144 PMC3832936

[bib25] Sashida G, Harada H, Matsui H, Oshima M, Yui M, Harada Y et al. Ezh2 loss promotes development of myelodysplastic syndrome but attenuates its predisposition to leukaemic transformation. Nat Commun 2014; 5: 4177. 24953053 10.1038/ncomms5177

[bib26] Mochizuki-Kashio M, Aoyama K, Sashida G, Oshima M, Tomioka T, Muto T et al. Ezh2 loss in hematopoietic stem cells predisposes mice to develop heterogeneous malignancies in an Ezh1-dependent manner. Blood 2015; 126: 1172–1183. 26219303 10.1182/blood-2015-03-634428

[bib27] Pratcorona M, Abbas S, Sanders MA, Koenders JE, Kavelaars FG, Erpelinck-Verschueren CA et al. Acquired mutations in ASXL1 in acute myeloid leukemia: prevalence and prognostic value. Haematologica 2012; 97: 388–392. 22058207 10.3324/haematol.2011.051532 PMC3291593

[bib28] Abdel-Wahab O, Adli M, LaFave LM, Gao J, Hricik T, Shih AH et al. ASXL1 mutations promote myeloid transformation through loss of PRC2-mediated gene repression. Cancer Cell 2012; 22: 180–193. 22897849 10.1016/j.ccr.2012.06.032 PMC3422511

[bib29] Dey A, Seshasayee D, Noubade R, French DM, Liu J, Chaurushiya MS et al. Loss of the tumor suppressor BAP1 causes myeloid transformation. Science 2012; 337: 1541–1546. 22878500 10.1126/science.1221711 PMC5201002

[bib30] Sahtoe DD, van Dijk WJ, Ekkebus R, Ovaa H, Sixma TK. BAP1/ASXL1 recruitment and activation for H2A deubiquitination. Nat Commun 2016; 7: 10292. 26739236 10.1038/ncomms10292 PMC4729829

[bib31] Abdel-Wahab O, Gao J, Adli M, Dey A, Trimarchi T, Chung YR et al. Deletion of Asxl1 results in myelodysplasia and severe developmental defects *in vivo*. J Exp Med 2013; 210: 2641–2659. 24218140 10.1084/jem.20131141 PMC3832937

[bib32] Puda A, Milosevic JD, Berg T, Klampfl T, Harutyunyan AS, Gisslinger B et al. Frequent deletions of JARID2 in leukemic transformation of chronic myeloid malignancies. Am J Hematol 2012; 87: 245–250. 22190018 10.1002/ajh.22257

[bib33] Meyer C, Hofmann J, Burmeister T, Groger D, Park TS, Emerenciano M et al. The MLL recombinome of acute leukemias in 2013. Leukemia 2013; 27: 2165–2176. 23628958 10.1038/leu.2013.135 PMC3826032

[bib34] Zeisig BB, Cheung N, Yeung J, So CW. Reconstructing the disease model and epigenetic networks for MLL-AF4 leukemia. Cancer Cell 2008; 14: 345–347. 18977321 10.1016/j.ccr.2008.10.008

[bib35] Guenther MG, Lawton LN, Rozovskaia T, Frampton GM, Levine SS, Volkert TL et al. Aberrant chromatin at genes encoding stem cell regulators in human mixed-lineage leukemia. Genes Dev 2008; 22: 3403–3408. 19141473 10.1101/gad.1741408 PMC2607073

[bib36] Nguyen AT, Taranova O, He J, Zhang Y. DOT1L the H3K79 methyltransferase, is required for MLL-AF9-mediated leukemogenesis. Blood 2011; 117: 6912–6922. 21521783 10.1182/blood-2011-02-334359 PMC3128482

[bib37] Chang MJ, Wu H, Achille NJ, Reisenauer MR, Chou CW, Zeleznik-Le NJ et al. Histone H3 lysine 79 methyltransferase Dot1 is required for immortalization by MLL oncogenes. Cancer Res 2010; 70: 10234–10242. 21159644 10.1158/0008-5472.CAN-10-3294 PMC3040779

[bib38] Bernt KM, Zhu N, Sinha AU, Vempati S, Faber J, Krivtsov AV et al. MLL-rearranged leukemia is dependent on aberrant H3K79 methylation by DOT1L. Cancer Cell 2011; 20: 66–78. 21741597 10.1016/j.ccr.2011.06.010 PMC3329803

[bib39] Okada Y, Feng Q, Lin Y, Jiang Q, Li Y, Coffield VM et al. hDOT1L links histone methylation to leukemogenesis. Cell 2005; 121: 167–178. 15851025 10.1016/j.cell.2005.02.020

[bib40] Deshpande AJ, Chen L, Fazio M, Sinha AU, Bernt KM, Banka D et al. Leukemic transformation by the MLL-AF6 fusion oncogene requires the H3K79 methyltransferase Dot1l. Blood 2013; 121: 2533–2541. 23361907 10.1182/blood-2012-11-465120 PMC3612861

[bib41] Cheung N, Chan LC, Thompson A, Cleary ML, So CW. Protein arginine-methyltransferase-dependent oncogenesis. Nat Cell Biol 2007; 9: 1208–1215. 17891136 10.1038/ncb1642

[bib42] Cheung N, Fung TK, Zeisig BB, K. H, Rane JK, Mowen KA et al. Targeting aberrant epigenetic networks mediated by PRMT1 and KDM4C in acute myeloid leukemia. Cancer Cell 2016; 29: 32–48. 26766589 10.1016/j.ccell.2015.12.007 PMC4712026

[bib43] Shia WJ, Okumura AJ, Yan M, Sarkeshik A, Lo MC, Matsuura S et al. PRMT1 interacts with AML1-ETO to promote its transcriptional activation and progenitor cell proliferative potential. Blood 2012; 119: 4953–4962. 22498736 10.1182/blood-2011-04-347476 PMC3367897

[bib44] Neff T, Sinha AU, Kluk MJ, Zhu N, Khattab MH, Stein L et al. Polycomb repressive complex 2 is required for MLL-AF9 leukemia. Proc Natl Acad Sci USA 2012; 109: 5028–5033. 22396593 10.1073/pnas.1202258109 PMC3324004

[bib45] Danis E, Yamauchi T, Echanique K, Haladyna J, Kalkur R, Riedel S et al. Inactivation of Eed impedes MLL-AF9-mediated leukemogenesis through Cdkn2a-dependent and Cdkn2a-independent mechanisms in a murine model. Exp Hematol 2015; 43: 930–935.e6. 26118502 10.1016/j.exphem.2015.06.005 PMC4630114

[bib46] Shi Y, Lan F, Matson C, Mulligan P, Whetstine JR, Cole PA et al. Histone demethylation mediated by the nuclear amine oxidase homolog LSD1. Cell 2004; 119: 941–953. 15620353 10.1016/j.cell.2004.12.012

[bib47] Klose RJ, Kallin EM, Zhang Y. JmjC-domain-containing proteins and histone demethylation. Nat Rev Genet 2006; 7: 715–727. 16983801 10.1038/nrg1945

[bib48] Harris WJ, Huang X, Lynch JT, Spencer GJ, Hitchin JR, Li Y et al. The histone demethylase KDM1A sustains the oncogenic potential of MLL-AF9 leukemia stem cells. Cancer Cell 2012; 21: 473–487. 22464800 10.1016/j.ccr.2012.03.014

[bib49] Wong SH, Goode DL, Iwasaki M, Wei MC, Kuo HP, Zhu L et al. The H3K4-methyl epigenome regulates leukemia stem cell oncogenic potential. Cancer Cell 2015; 28: 198–209. 26190263 10.1016/j.ccell.2015.06.003 PMC4536132

[bib50] Schenk T, Chen WC, Gollner S, Howell L, Jin L, Hebestreit K et al. Inhibition of the LSD1 (KDM1A) demethylase reactivates the all-trans-retinoic acid differentiation pathway in acute myeloid leukemia. Nat Med 2012; 18: 605–611. 22406747 10.1038/nm.2661 PMC3539284

[bib51] He J, Nguyen AT, Zhang Y. KDM2b/JHDM1b, an H3K36me2-specific demethylase, is required for initiation and maintenance of acute myeloid leukemia. Blood 2011; 117: 3869–3880. 21310926 10.1182/blood-2010-10-312736 PMC3083299

[bib52] Sroczynska P, Cruickshank VA, Bukowski JP, Miyagi S, Bagger FO, Walfridsson J et al. shRNA screening identifies JMJD1C as being required for leukemia maintenance. Blood 2014; 123: 1870–1882. 24501218 10.1182/blood-2013-08-522094

[bib53] Chen M, Zhu N, Liu X, Laurent B, Tang Z, Eng R et al. JMJD1C is required for the survival of acute myeloid leukemia by functioning as a coactivator for key transcription factors. Genes Dev 2015; 29: 2123–2139. 26494788 10.1101/gad.267278.115 PMC4617977

[bib54] Zhu N, Chen M, Eng R, DeJong J, Sinha AU, Rahnamay NF et al. MLL-AF9- and HOXA9-mediated acute myeloid leukemia stem cell self-renewal requires JMJD1C. J Clin Invest 2016; 126: 997–1011. 26878175 10.1172/JCI82978 PMC4767347

[bib55] Chen CW, Koche RP, Sinha AU, Deshpande AJ, Zhu N, Eng R et al. DOT1L inhibits SIRT1-mediated epigenetic silencing to maintain leukemic gene expression in MLL-rearranged leukemia. Nat Med 2015; 21: 335–343. 25822366 10.1038/nm.3832 PMC4390532

[bib56] Agger K, Miyagi S, Pedersen MT, Kooistra SM, Johansen JV, Helin K. Jmjd2/Kdm4 demethylases are required for expression of Il3ra and survival of acute myeloid leukemia cells. Genes Dev 2016; 30: 1278–1288. 27257215 10.1101/gad.280495.116 PMC4911927

[bib57] Arteaga MF, Mikesch JH, Qiu J, Christensen J, Helin K, Kogan SC et al. The histone demethylase PHF8 governs retinoic acid response in acute promyelocytic leukemia. Cancer Cell 2013; 23: 376–389. 23518351 10.1016/j.ccr.2013.02.014 PMC6812572

[bib58] Fung TK, So CW. Overcoming treatment resistance in acute promyelocytic leukemia and beyond. Oncotarget 2013; 4: 1128–1129. 23934744 10.18632/oncotarget.1244 PMC3787144

[bib59] Daigle SR, Olhava EJ, Therkelsen CA, Majer CR, Sneeringer CJ, Song J et al. Selective killing of mixed lineage leukemia cells by a potent small-molecule DOT1L inhibitor. Cancer Cell 2011; 20: 53–65. 21741596 10.1016/j.ccr.2011.06.009 PMC4046888

[bib60] Daigle SR, Olhava EJ, Therkelsen CA, Basavapathruni A, Jin L, Boriack-Sjodin PA et al. Potent inhibition of DOT1L as treatment of MLL-fusion leukemia. Blood 2013; 122: 1017–1025. 23801631 10.1182/blood-2013-04-497644 PMC3739029

[bib61] Zhou J, Bi C, Cheong LL, Mahara S, Liu SC, Tay KG et al. The histone methyltransferase inhibitor, DZNep, up-regulates TXNIP, increases ROS production, and targets leukemia cells in AML. Blood 2011; 118: 2830–2839. 21734239 10.1182/blood-2010-07-294827

[bib62] Neff T, Sinha AU, Kluk MJ, Zhu N, Khattab MH, Stein L et al. Polycomb repressive complex 2 is required for MLL-AF9 leukemia. Proc Natl Acad Sci USA 2012; 109: 5028–5033. 22396593 10.1073/pnas.1202258109 PMC3324004

[bib63] Xu B, On DM, Ma A, Parton T, Konze KD, Pattenden SG et al. Selective inhibition of EZH2 and EZH1 enzymatic activity by a small molecule suppresses MLL-rearranged leukemia. Blood 2015; 125: 346–357. 25395428 10.1182/blood-2014-06-581082 PMC4287641

[bib64] Knutson SK, Wigle TJ, Warholic NM, Sneeringer CJ, Allain CJ, Klaus CR et al. A selective inhibitor of EZH2 blocks H3K27 methylation and kills mutant lymphoma cells. Nat Chem Biol 2012; 8: 890–896. 23023262 10.1038/nchembio.1084

[bib65] McCabe MT, Ott HM, Ganji G, Korenchuk S, Thompson C, Van Aller GS et al. EZH2 inhibition as a therapeutic strategy for lymphoma with EZH2-activating mutations. Nature 2012; 492: 108–112. 23051747 10.1038/nature11606

[bib66] Kim W, Bird GH, Neff T, Guo G, Kerenyi MA, Walensky LD et al. Targeted disruption of the EZH2-EED complex inhibits EZH2-dependent cancer. Nat Chem Biol 2013; 9: 643–650. 23974116 10.1038/nchembio.1331 PMC3778130

[bib67] Gahr M, Schonfeldt-Lecuona C, Kolle MA, Freudenmann RW. Intoxications with the monoamine oxidase inhibitor tranylcypromine: an analysis of fatal and non-fatal events. Eur Neuropsychopharmacol 2013; 23: 1364–1372. 23791433 10.1016/j.euroneuro.2013.05.009

[bib68] Fiskus W, Sharma S, Shah B, Portier BP, Devaraj SG, Liu K et al. Highly effective combination of LSD1 (KDM1A) antagonist and pan-histone deacetylase inhibitor against human AML cells. Leukemia 2014; 28: 2155–2164. 24699304 10.1038/leu.2014.119 PMC4739780

[bib69] Wysocka J, Swigut T, Milne TA, Dou Y, Zhang X, Burlingame AL et al. WDR5 associates with histone H3 methylated at K4 and is essential for H3 K4 methylation and vertebrate development. Cell 2005; 121: 859–872. 15960974 10.1016/j.cell.2005.03.036

[bib70] Ruthenburg AJ, Wang W, Graybosch DM, Li H, Allis CD, Patel DJ et al. Histone H3 recognition and presentation by the WDR5 module of the MLL1 complex. Nat Struct Mol Biol 2006; 13: 704–712. 16829959 10.1038/nsmb1119 PMC4698793

[bib71] Cao F, Townsend EC, Karatas H, Xu J, Li L, Lee S et al. Targeting MLL1 H3K4 methyltransferase activity in mixed-lineage leukemia. Mol Cell 2014; 53: 247–261. 24389101 10.1016/j.molcel.2013.12.001 PMC3965208

[bib72] Dawson MA, Prinjha RK, Dittmann A, Giotopoulos G, Bantscheff M, Chan WI et al. Inhibition of BET recruitment to chromatin as an effective treatment for MLL-fusion leukaemia. Nature 2011; 478: 529–533. 21964340 10.1038/nature10509 PMC3679520

[bib73] Zuber J, Shi J, Wang E, Rappaport AR, Herrmann H, Sison EA et al. RNAi screen identifies Brd4 as a therapeutic target in acute myeloid leukaemia. Nature 2011; 478: 524–528. 21814200 10.1038/nature10334 PMC3328300

[bib74] Fong CY, Gilan O, Lam EY, Rubin AF, Ftouni S, Tyler D et al. BET inhibitor resistance emerges from leukaemia stem cells. Nature 2015; 525: 538–542. 26367796 10.1038/nature14888 PMC6069604

[bib75] Rathert P, Roth M, Neumann T, Muerdter F, Roe JS, Muhar M et al. Transcriptional plasticity promotes primary and acquired resistance to BET inhibition. Nature 2015; 525: 543–547. 26367798 10.1038/nature14898 PMC4921058

